# The Pilot Study of Evaluating Fluctuation in the Blood Flow Volume of the Radial Artery, a Site for Traditional Pulse Diagnosis

**DOI:** 10.3390/medicines3020011

**Published:** 2016-05-17

**Authors:** Masashi Watanabe, Soichiro Kaneko, Shin Takayama, Yasuyuki Shiraishi, Takehiro Numata, Natsumi Saito, Takashi Seki, Norihiro Sugita, Satoshi Konno, Tomoyuki Yambe, Makoto Yoshizawa, Nobuo Yaegashi, Tadashi Ishii

**Affiliations:** 1 Department of Education and Support for Regional Medicine, Department of Kampo Medicine, Tohoku University Hospital, 1-1 Seiryou-machi, Aoba-ku, Sendai, Miyagi 980-8574, Japan; egao.2008@wine.ocn.ne.jp (M.W.); souichi0134@gmail.com (S.K.); numatatakehiro@gmail.com (T.N.); natsu.beauty.summer@gmail.com (N.S.); t-ishi23@green.ocn.ne.jp (T.I.); 2 Comprehensive Education Center for Community Medicine, Graduate School of Medicine, Tohoku University, 2-1 Seiryou-machi, Aoba-ku, Sendai, Miyagi 980-8575, Japan; 3 Institute of Development, Aging and Cancer, Tohoku University, 4-1 Seiryo-machi, Aoba-ku, Sendai, Miyagi 980-8575, Japan; shiraishi@idac.tohoku.ac.jp (Y.S.); konnos@idac.tohoku.ac.jp (S.K.); yambe@idac.tohoku.ac.jp (T.Y.); 4 Division of Cyclotron Nuclear Medicine, Cyclotron and Radioisotope Center, Tohoku University, 6-3 Aoba, Aramaki, Aoba-ku, Sendai, Miyagi 980-8578, Japan; tseki.tohoku@gmail.com; 5 Department of Management Science and Technology, Graduate School of Engineering, Tohoku University, 6-6-05 Aramaki Aza Aoba, Aoba-ku, Sendai, Miyagi 980-8579, Japan; sugita@yoshizawa.ecei.tohoku.ac.jp; 6 Research Division on Advanced Information Technology, Cyberscience Center, Tohoku University, 6-3 Aramaki Aza Aoba, Aoba-ku, Sendai, Miyagi 980-8578, Japan; yoshizawa@yoshizawa.ecei.tohoku.ac.jp; 7 Department of Obstetrics and Gynecology, Graduate School of Medicine, Tohoku University, 1-1 Seiryou-machi, Aoba-ku, Sendai, Miyagi 980-8574, Japan; yaegashi@med.tohoku.ac.jp

**Keywords:** pulse diagnosis, radial artery, blood flow volume, fluctuation, ultrasonography

## Abstract

Background: Radial artery (RA) pulse diagnosis has been used in traditional Asian medicine. Blood pressure (BP) and pulse rate related to heart rate variability (HRV) can be monitored via the RA. The fluctuation in these parameters has been assessed using fast Fourier transform (FFT) analytical methods that calculate power spectra. Methods: We measured blood flow volume (Volume) in the RA and evaluated its fluctuations. Normal participants (*n* = 34) were enrolled. We measured the hemodynamics of the right RA for approximately 50 s using ultrasonography. Results: The parameters showed the center frequency (CF) of the power spectrum at low frequency (LF) and high frequency (HF). More than one spectral component indicated that there were fluctuations. The CF at LF for Volume was significantly different from that for vessel diameter (VD); however, it was significantly correlated with blood flow velocity (Velocity). On the other hand, the CF at HF for Volume was significantly different from that for Velocity; however, it was significantly correlated with VD. Conclusion: It is suggested that fluctuation in the Volume at LF of RA is influenced by the fluctuation in Velocity; on the other hand, fluctuation in the Volume at HF is influenced by the fluctuation in VD.

## 1. Introduction

Pulse diagnosis has been used in traditional medicine since ancient times (e.g., traditional Chinese medicine [1], Ayurveda [2], Unani medicine [3] and Tibetan medicine [4,5,6,7]). It is performed on the radial artery in the area around the radial styloid process, carotid artery and other arteries [8]. Traditional pulse diagnosis depends on experience, and quantification is very difficult. Therefore, it is difficult to carry out by education in Western medicine. Recently, a technique to measure a pulse wave was developed. Additionally, it is expected that various things become clear by evaluating the fluctuation included in the pulse wave [9].

Blood pressure (BP) and heart rate (HR) related to heart rate variability (HRV) can be monitored via the radial artery [10]. Fluctuations in the HR and BP have been assessed using fast Fourier transform (FFT) analytical methods that calculate power spectra [11,12,13,14,15]. Low frequency (LF) and high frequency (HF) components are observed in the spectrum of HRV [14,16,17]. The LF fluctuations in HRV are mediated by both the sympathetic and parasympathetic nervous systems, whereas the HF fluctuations are mediated solely by the parasympathetic system [14]. The blood flow volume is calculated using the vascular diameter (VD) and blood flow velocity (velocity). VD and velocity are evaluated using ultrasonic diagnostic equipment. VD is affected by the fluid content and through neurogenic control. The cell membranes of the smooth muscle cells in the vessels contain α- and β-adrenergic receptors that bind to neurotransmitters. Activation of α-adrenergic receptors promotes vasoconstriction, while the activation of β-adrenergic receptors mediates the relaxation of muscle cells, resulting in vasodilation. Normally, α-adrenergic receptors predominate in the smooth muscle of resistance vessels. Thus, vascular smooth muscle is ruled out of the sympathetic nerve-mediated tonicity. In addition, there are few reports about vasomotor fluctuation [18,19]. However, there are no studies yet that have measured the variability of the blood flow volume (Volume) in the radial artery. We measured the Volume in the radial artery and evaluated its fluctuations at the site of traditional pulse diagnosis using a high-resolution ultrasonography in the participants at rest.

The purpose of the pilot study was to measure the blood flow in the pulse diagnosis site and to explore its fluctuation related to the physiological parameters.

## 2. Experimental Section

### 2.1. Subjects

Thirty-three healthy volunteers (26 men and 7 women; mean age 34.2 ± 7.6 years) were enrolled in the study. All participants provided written informed consent before participation, and the study protocol was approved by the Ethics Committee of Tohoku University Graduate School of Medicine, Project identification code (2009-175) and approval date (27 July 2009) (Table 1).

### 2.2. Study Protocol

**Figure 1:**
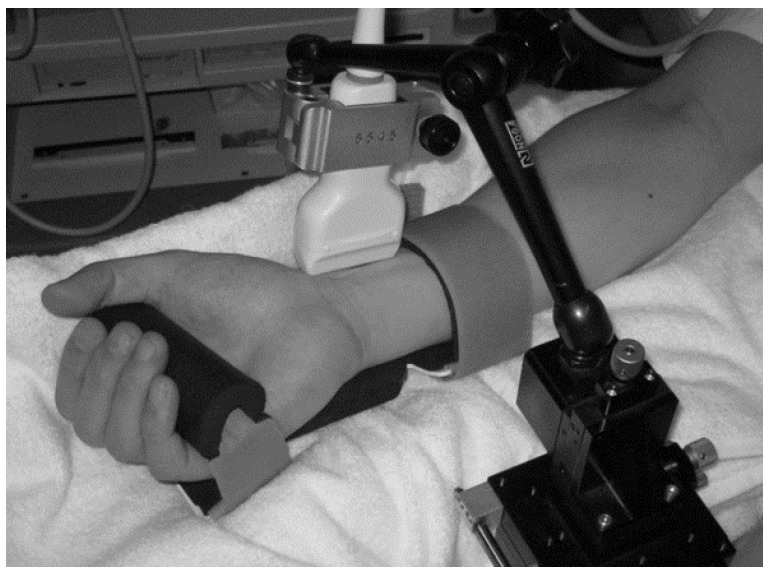
Ultrasonography measurement of radial artery using a special probe holder.

**Figure 2:**
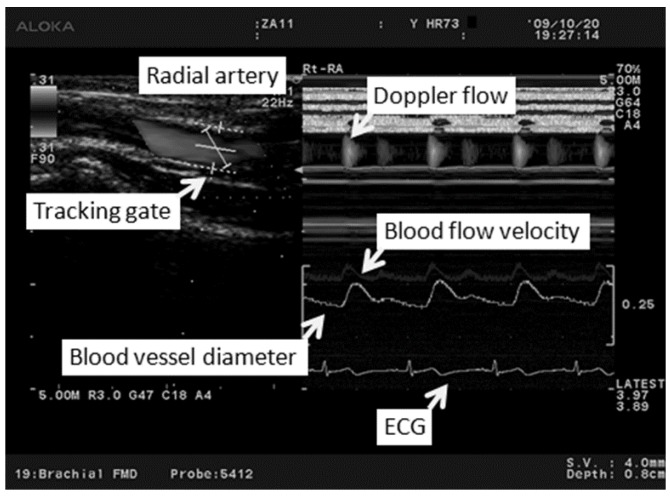
The image on the left shows the radial artery and the position of the tracking gate on the artery. The image on the right shows the changes in the vessel diameter, Doppler flow and flow velocity determined using an automated edge detection device and computer analysis software.

All investigations were performed under fasting conditions in a quiet air-conditioned room (constant temperature of 25–26 °C). Each participant was at rest in the supine position, and three electrocardiography electrodes (BP-608 Evolution II, Colin Healthcare Co. Ltd., Kyoto, Japan) were attached to the chest for monitoring. Radial artery hemodynamics were assessed using ultrasonography (Prosound α10^®^; Hitachi-Aloka Medical, Ltd., Tokyo, Japan). This system had a high-resolution linear array transducer (13 MHz, Prosound α10^®^; Hitachi-Aloka Medical, Ltd., Tokyo, Japan) and computer-assisted analysis software (e-Tracking system^®^; Hitachi-Aloka Medical, Ltd., Tokyo, Japan) that could automatically detect a blood vessel edge and continuously measure the vessel diameter and Volume [20]. The right arm was fixed and the right radial artery was scanned longitudinally at 1–2 cm above the radial styloid process at a point where the vessel diameter and Doppler wave readings were stable. At a site where the clearest B-mode image of the anterior and posterior intimal interfaces between the lumen and vessel wall was obtained, the transducer was fixed in place using a special probe holder (MP-PH0001; Hitachi-Aloka Medical, Ltd., Tokyo, Japan). Compression of the artery was carefully avoided (Figure 1). When the tracking gate was placed on the intima, the radial artery diameter was monitored automatically, and a waveform representing the changes of vessel diameter during a cardiac cycle was displayed in real time using the e-Tracking system^®^ (Figure 2). To obtain accurate measurements, a Doppler angle of ≤60 degrees was maintained [21,22]. Volume was calculated automatically as the Doppler flow velocity (corrected for the angle) multiplied by the vessel cross-sectional area [21,22,23]. We measured right radial artery hemodynamics for approximately 50 s after 10 min of rest in the supine position [24,25]. The hemodynamic parameters, including the radial artery diameter and Volume, and the HR were recorded continuously. To minimize the influence of respiration on the hemodynamic data, the participants were asked to breathe every 6 s during the test. Measured parameters were systolic vessel diameter (Sys-VD) per beat, diastolic vessel diameter (Dia-VD) per beat, Velocity per second, volume per beat and HR per minute.

### 2.3. Analysis

In order to produce an evenly-sampled time series prior to FFT-based spectral estimation, linear spline interpolation and resampling of measured parameter data are usually employed [26,27]. After removal of linear trends included in the data, FFT analysis was applied using MATLAB software (Version 2007b; MathWorks, Natick, MA, USA) to obtain power spectra (periodogram) for Sys-VD, Dia-VD, HR, Velocity and Volume. In a low frequency component (LF; 0.04–0.15 Hz), we compared each of the center frequencies (CF) as follows: (1) Volume with Sys-VD; (2) Volume with Dia-VD; and (3) Volume with Velocity. In a high frequency component (HF; <0.15 Hz), we compared each of the CF as follows: (1) Volume with Sys-VD; (2) Volume with Dia-VD; and (3) Volume with Velocity.

### 2.4. Statistical Methods

Statistical analyses were performed using the PASW software (Version 17.0, SPSS Japan Inc., Tokyo, Japan). Comparisons between parameters were performed using ANOVA with Dunnett’s *post hoc* tests and Pearson’s product-moment correlation coefficient. Statistical significance was assumed at *p* < 0.05. Values are the mean ± standard deviation (SD).

## 3. Results

**Figure 3:**
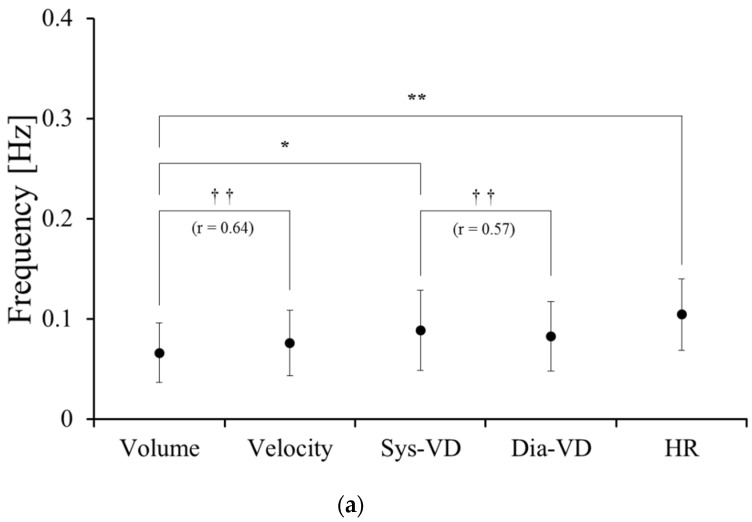

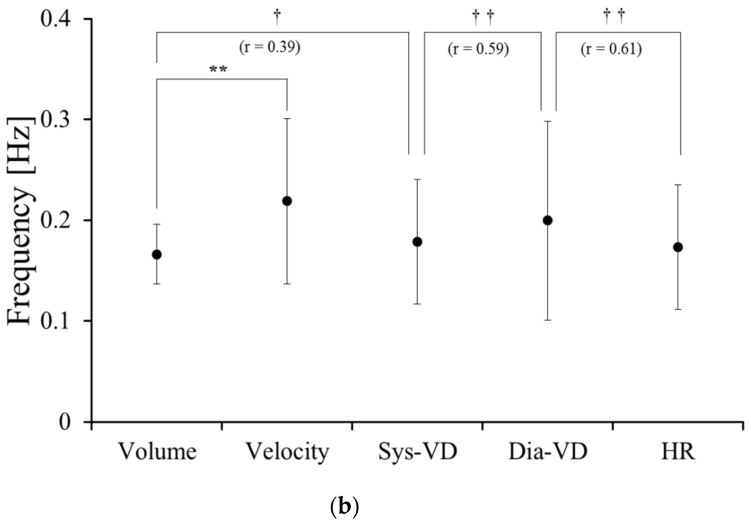
Comparison among the center frequency of blood flow volume (Volume), blood flow velocity (Velocity), systolic vessel diameter (Sys-VD), diastolic vessel diameter (Dia-VD) and heart rate (HR) in (**a**) the low-frequency component (LF) and (**b**) the high-frequency component (HF). Values represent the mean ± standard deviation (SD). Comparisons between parameters were performed using ANOVA with Dunnett’s *post hoc* tests (* *p* < 0.05; ** *p* < 0.01) and Pearson’s product-moment correlation coefficient (*r*: correlation coefficient, † *p* < 0.05; †† *p* < 0.01). Statistical significance was assumed at *p* < 0.05.

In all participants, the CF was seen in the LF and HF (Table 2). Comparisons of the CF of the LF and HF are shown in Figure 3a,b, respectively. There were significant differences in the CF between (1) the LF in Volume and the LF in HR (*p* < 0.01) and (2) the LF in Volume and the LF in Sys-VD (*p* < 0.05). There were significant correlate in the CF between (3) the LF in Volume and the LF in Velocity (*r* = 0.64, *p* < 0.01) and (4) the LF in Sys-VD and the LF in Dia-VD (*r* = 0.57, *p* < 0.01). On the other hand, there were significant differences in the CF between (5) the HF in Volume and the HF in Velocity (*p* < 0.01). There were significant correlate in the CF between (6) the HF in Volume and the HF in Sys-VD (*r* = 0.39, *p* < 0.05), (7) the HF in Sys-VD and the HF in Dia-VD (*r* = 0.59, *p* < 0.01) and (8) the HF in Dia-VD and the HF in HR (*r* = 0.61, *p* < 0.01).

## 4. Discussion

A peak appears in the spectrum only due to the superposition of a number of waves from a time series comprising multiple waves [28]. HRV has different LF and HF that are observed in a spectrum for individual peaks [28]. In our analysis, we observed LF and HF in Sys-VD, Dia-VD, HR, Velocity and Volume in the radial artery while subjects were at rest. More than one spectral component indicated that there were fluctuations in each of these parameters. In a previous study on fluctuations in the circulatory system, only HRV and BP were studied [10,13,16,17,28,29,30,31,32,33,34], but there has been no report on Volume of the peripheral artery. In this study, to our knowledge, this is the first report of the investigation of the fluctuations in Volume of the radial artery. Frequencies of 0.085 and 0.09 Hz have been reported as the CF in the LF, and those of 0.21 and 0.24 Hz have been reported as the CF in the HF [17,31]. The LF appears to correspond to Mayer waves, while the HF is synchronous with respiration and has been considered as a quantitative evaluation of respiratory arrhythmia [17]. The HF varies in its CF with variations in the respiratory cycle [35]. In this study, the participants were asked to breathe every 6 s during the test, and this could have influenced the HF. The frequency during breathe control is 0.167 Hz, which was equivalent to that of the HF (0.166 Hz) in our results. It will be necessary to repeat the study without breath control. According to our results, in LF, there was a significant difference in the CF between Volume and HR/vessel diameter, while there was significant correlation between Volume and Velocity. In HF, there was a significant difference in the CF between Volume and Velocity, while there was significant correlation between Volume and vessel diameter. These results show that the CF of the Volume in LF is correlated with the CF of the Velocity in LF; on the other hand, the CF of the Volume in HF is correlated with the CF of the vessel diameter in HF.

In peripheral arteries, such as radial arteries, there are influences of local vasoconstrictor and vasodilator nerves, as well as the influence of the autonomic nerves on the heart. There are differences with regard to the LF. Some studies suggest that LF is a quantitative marker of sympathetic modulations, while other studies view LF as reflecting both sympathetic and vagal activities. Consequently, an LF/HF ratio is considered by some investigators to mirror a sympathovagal balance or to reflect sympathetic modulations [30]. In this study, the value of the LF/HF ratio for the Volume was 2.10. According to the previous reports, this value shows a rest state, but there is a difference by some reports [36,37,38,39].

Investigators from the Framingham Heart Study reported that HRV offers prognostic information independent of and beyond that provided by traditional risk factors [30]. The fluctuations of the LF/HF ratio in Volume of the radial artery could be a new index for the prognosis of cardiac vascular disease. The CF of the LF in Volume of the radial artery indicates a cycle of 7–25 s. This suggests that more than 25 s might be required for pulse diagnosis.

At the site of a pulse diagnosis using a radial artery, the blood vessel diameter is so small that the instrument used for measurement points can slip off due to slight movements by the participants. Therefore, in this study, it was difficult to make measurements for prolonged periods. For future investigations, an easier, more stable method of measurement must be established. The limitation of this study is also a lack of measurements of continuous BP and the sampling duration. To evaluate the relation among the HR, BP and blood flow, simultaneous measurements of these parameters are needed. We have conducted a further study that solves these problems and the evaluation introduced to investigate acupuncture effects. The duration of blood flow sampling used for analysis is a little shorter than ideal. Generally, the spectral analysis requires over 60 s of data. In the present study, the arms of the subjects were fixed to measure blood flow using ultrasonography; however, occasionally, the subjects moved their arms during long duration measurements. We have tried analyzing over 60 s of data for spectral analysis; unfortunately, error is introduced once the subjects move their arm. Therefore, we used data for a shorter period (50 s), which was free of errors. Thus, the sampling duration is one of the limitations of this study. The present study is a pilot study; therefore, we will conduct experiments using a revised protocol in the future. We also intend to increase the number of subjects and to perform these studies with considerations of gender, age and disease. Further investigation will be needed for healthy subjects and patients with cardiovascular disease to show the clinical difference and meanings.

## 5. Conclusions

We measured the blood flow volume in the radial artery and evaluated its fluctuations at the site of traditional pulse diagnosis using ultrasonography in the participants at rest. We observed LF and HF in vessel diameter, HR, Velocity and Volume. More than one spectral component indicated that there were fluctuations in each of these parameters. This study showed that the CF of the Volume in LF was correlated with CF of the Velocity in LF, on the other hand, the CF of the Volume in HF was correlated with CF of the vessel diameter in HF.

## Figures and Tables

**Table 1:** Participant’s characteristics for this study.

Characteristic		Variable
Age (*y*)		34.2 ± 7.6
Sex (*n*)	Male	26
	Female	7
Body height (cm)		168.4 ± 6.7
Body weight (kg)		67.0 ± 13.0

**Table 2:** Center frequencies (Hz) and low frequency (LF)/high frequency (HF) ratio in the LF component and HF component of all subjects in blood flow volume (Volume), blood flow velocity (Velocity), systolic vessel diameter (Sys-VD), diastolic vessel diameter (Dia-VD) and heart rate (HR).

Participant	Volume		Velocity		Sys-VD		Dia-VD		HR	
No.	LF (Hz)	HF (Hz)	LF (Hz)	HF (Hz)	LF (Hz)	HF (Hz)	LF (Hz)	HF (Hz)	LF (Hz)	HF (Hz)
1	0.137	0.156	0.137	0.156	0.049	0.156	0.049	0.166	0.049	0.146
2	0.049	0.166	0.049	0.166	0.137	0.176	0.049	0.156	0.068	0.166
3	0.137	0.166	0.137	0.156	0.137	0.166	0.049	0.166	0.137	0.166
4	0.049	0.156	0.098	0.166	0.059	0.166	0.068	0.146	0.059	0.166
5	0.059	0.166	0.059	0.166	0.049	0.156	0.049	0.156	0.059	0.156
6	0.078	0.156	0.088	0.186	0.107	0.156	0.049	0.156	0.137	0.146
7	0.059	0.234	0.059	0.313	0.078	0.273	0.078	0.225	0.068	0.156
8	0.078	0.146	0.088	0.410	0.137	0.146	0.137	0.146	0.137	0.146
9	0.049	0.146	0.088	0.146	0.137	0.156	0.049	0.488	0.137	0.146
10	0.049	0.303	0.049	0.293	0.078	0.264	0.088	0.332	0.088	0.273
11	0.049	0.146	0.049	0.303	0.049	0.176	0.088	0.488	0.049	0.498
12	0.049	0.156	0.059	0.332	0.049	0.156	0.049	0.146	0.127	0.146
13	0.049	0.146	0.117	0.342	0.049	0.146	0.049	0.146	0.137	0.146
14	0.049	0.176	0.049	0.215	0.088	0.488	0.078	0.498	0.088	0.195
15	0.049	0.166	0.049	0.166	0.137	0.156	0.137	0.156	0.137	0.156
16	0.059	0.156	0.059	0.156	0.137	0.166	0.117	0.166	0.059	0.166
17	0.049	0.156	0.049	0.322	0.137	0.166	0.088	0.166	0.068	0.166
18	0.049	0.146	0.049	0.371	0.049	0.215	0.049	0.166	0.137	0.146
19	0.049	0.166	0.049	0.244	0.049	0.146	0.117	0.156	0.049	0.166
20	0.049	0.166	0.107	0.166	0.049	0.166	0.049	0.166	0.137	0.166
21	0.059	0.166	0.068	0.166	0.137	0.166	0.098	0.166	0.137	0.156
22	0.049	0.166	0.078	0.361	0.137	0.156	0.137	0.156	0.117	0.166
23	0.098	0.176	0.127	0.176	0.049	0.166	0.098	0.166	0.088	0.166
24	0.068	0.166	0.068	0.166	0.098	0.166	0.107	0.176	0.049	0.166
25	0.137	0.166	0.137	0.166	0.049	0.166	0.049	0.166	0.137	0.166
26	0.049	0.146	0.049	0.205	0.049	0.166	0.049	0.156	0.137	0.146
27	0.049	0.195	0.049	0.146	0.049	0.146	0.049	0.244	0.078	0.156
28	0.049	0.166	0.049	0.166	0.049	0.166	0.049	0.166	0.137	0.166
29	0.127	0.146	0.049	0.166	0.137	0.166	0.137	0.166	0.137	0.156
30	0.059	0.146	0.137	0.166	0.127	0.186	0.127	0.186	0.078	0.186
31	0.117	0.146	0.127	0.146	0.127	0.146	0.127	0.146	0.117	0.146
32	0.049	0.146	0.049	0.146	0.137	0.156	0.137	0.156	0.137	0.156
33	0.049	0.166	0.068	0.166	0.049	0.166	0.049	0.166	0.137	0.166
34	0.049	0.166	0.049	0.322	0.068	0.156	0.107	0.176	0.137	0.166
Mean ± SD	0.066 ± 0.030	0.166 ± 0.030	0.076 ± 0.033	0.219 ± 0.082	0.089 ± 0.040	0.179 ± 0.062	0.082 ± 0.035	0.200 ± 0.098	0.104 ± 0.036	0.173 ± 0.062
LF/HF Mean ± SD	2.1 ± 1.6		0.9 ± 0.5		0.8 ± 0.5		0.9 ± 0.5		0.8 ± 0.4	
